# Association of the CHA_2_D(S_2_)-VASc Score and Its Components With Overt and Silent Ischemic Brain Lesions in Patients With Atrial Fibrillation

**DOI:** 10.3389/fneur.2020.609234

**Published:** 2021-01-12

**Authors:** Fabienne Steiner, Pascal B. Meyre, Stefanie Aeschbacher, Michael Coslovsky, Tim Sinnecker, Manuel R. Blum, Nicolas Rodondi, Carlo W. Cereda, Marcello di Valentino, Florence Wenger, Andrea Cussigh, Philipp Krisai, Laurent Roten, Tobias Reichlin, David Conen, Stefan Osswald, Leo H. Bonati, Michael Kühne, The Swiss-AF Investigators

**Affiliations:** ^1^Cardiovascular Research Institute Basel, University Hospital Basel, University of Basel, Basel, Switzerland; ^2^Department of Cardiology, Department of Medicine, University Hospital Basel, University of Basel, Basel, Switzerland; ^3^Clinical Trial Unit Basel, Department of Clinical Research, University Hospital Basel, Basel, Switzerland; ^4^Medical Image Analysis Center (MIAC AG) and Department of Biomedical Engineering, University of Basel, Basel, Switzerland; ^5^Department of Neurology and Stroke Center, University Hospital Basel, University of Basel, Basel, Switzerland; ^6^Institute of Primary Health Care (BIHAM, Berner Institut für Hausarztmedizin), University of Bern, Bern, Switzerland; ^7^Department of General Medicine, Inselspital, Bern University Hospital, University of Bern, Bern, Switzerland; ^8^Neurocenter of Southern Switzerland, Neurology, Ospedale Regionale di Lugano, Lugano, Switzerland; ^9^Department of Cardiology, Ospedale San Giovanni, Bellinzona, Switzerland; ^10^Department of Cardiology, Inselspital, Bern University Hospital, University of Bern, Bern, Switzerland; ^11^Population Health Research Institute, McMaster University, Hamilton, ON, Canada

**Keywords:** atrial fibrillation, silent ischemic brain lesion, stroke, CHA_2_DS_2_-VASc score, ischemic brain lesion

## Abstract

**Background:** Silent and overt ischemic brain lesions are common and associated with adverse outcome. Whether the CHA_2_DS_2_-VASc score and its components predict magnetic resonance imaging (MRI)-detected ischemic silent and overt brain lesions in patients with atrial fibrillation (AF) is unclear.

**Methods:** In this cross-sectional analysis, patients with AF were enrolled in a multicenter cohort study in Switzerland. Outcomes were clinically overt, silent [in the absence of a history of stroke/transient ischemic attack (TIA)] and any MRI-detected ischemic brain lesions. Logistic regression analyses were performed to assess the relationship of the CHA_2_DS_2_-VASc score and its components with ischemic brain lesions. An adapted CHA_2_D-VASc score (excluding history of stroke/TIA) for the analyses of clinically overt and silent ischemic brain lesions was used.

**Results:** Overall, 1,741 patients were included in the analysis (age 73 ± 8 years, 27.4% female). At least one ischemic brain lesion was observed in 36.8% (clinically overt: 10.5%; silent: 22.9%; transient ischemic attack: 3.4%). The CHA_2_D-VASc score was strongly associated with clinically overt and silent ischemic brain lesions {odds ratio (OR) [95% confidence interval (CI)] 1.32 (1.17–1.49), *p* < 0.001 and 1.20 (1.10–1.30), *p* < 0.001, respectively}. Age 65–74 years (OR 2.58; 95%CI 1.29–5.90; *p* = 0.013), age ≥75 years (4.13; 2.07–9.43; *p* < 0.001), hypertension (1.90; 1.28–2.88; *p* = 0.002) and diabetes (1.48; 1.00–2.18; *p* = 0.047) were associated with clinically overt brain lesions, whereas age 65–74 years (1.95; 1.26–3.10; *p* = 0.004), age ≥75 years (3.06; 1.98–4.89; *p* < 0.001) and vascular disease (1.39; 1.07–1.79; *p* = 0.012) were associated with silent ischemic brain lesions.

**Conclusions:** A higher CHA_2_D-VASc score was associated with a higher risk of both overt and silent ischemic brain lesions.

**Clinical Trial Registration:** www.ClinicalTrials.gov, identifier: NCT02105844.

## Introduction

Patients with atrial fibrillation (AF) have a high risk of stroke, heart failure and death (1–3). Recent evidence suggests that patients with AF have a substantial burden of silent brain infarcts, detected only by magnetic resonance imaging (MRI) (4, 5). In a contemporary cohort of AF patients, the prevalence of silent infarcts was 25.7%. Currently, there is only limited data on risk factors for silent ischemic brain lesions in this group of patients (4). Since silent ischemic brain lesions are highly prevalent and have been reported to be strongly related with cognitive dysfunction and an increased risk of future symptomatic stroke (6), a better understanding of the potential risk factors is crucial for prevention and possible treatment strategies (4, 7).

The CHA_2_DS_2_-VASc score is an aggregate of modifiable and non-modifiable risk factors and has been developed to predict the individual stroke risk in patients with AF and to aid in the decision whether oral anticoagulation is warranted (8, 9). Although the CHA_2_DS_2_-VASc score is a commonly used clinical tool to risk stratify AF patients for future strokes, it is unclear whether and to what extent the score and its individual components are associated with the presence of imaging detected, clinically overt or silent ischemic brain lesions in patients with AF. Identifying the individual modifiable risk factors for silent ischemic brain lesions in AF patients may be relevant in order to take preventive measures at an early stage and improve the prevention of stroke and its sequelae in AF patients. Additionally, the predictive performance of the CHA_2_DS_2_-VASc score to detect ischemic silent or overt brain lesions is not yet known.

Therefore, the aim of the current study was to investigate the association and the predictive value of the CHA_2_DS_2_-VASc score, and specifically its individual components, with clinically overt or silent ischemic brain lesions detected on MRI, in a large cohort of AF patients mainly on oral anticoagulation.

## Methods

### Study Population

The analysis is based on data from the Swiss Atrial Fibrillation Cohort (Swiss-AF), an ongoing prospective, observational, multicenter cohort study. Overall, 2,415 patients with documented AF were enrolled between 2014 and 2017 across 14 centers in Switzerland. The main inclusion criteria were previously documented AF and age ≥65 years (a subgroup of 10% was aged between 45 and 65 years). Patients were excluded if they had short secondary, reversible episodes of AF (e.g., after cardiac surgery), had an acute illness within the last 4 weeks or were unable to sign informed consent (10). The study protocol has been approved by the local ethic committees, and written informed consent was obtained from each participant.

Of the 2,415 patients enrolled, 674 (27.9%) were excluded due to missing brain MRI (*n* = 667, 27.6%), or missing data on ischemic brain lesions at baseline visit (*n* = 5, 0.2%), or missing information on the CHA_2_DS_2_-VASc score (*n* = 2, 0.1%), resulting in 1,741 patients (72.1%) for this analysis (Supplementary Figure 1). The main reasons for not performing brain MRI were the presence of a cardiac implantable electronic device and claustrophobia.

### Study Procedures

Information on individual patient characteristics, medical history and current medication were collected using standardized case report forms. Body height and weight were measured at baseline, and body mass index (BMI) was calculated by dividing weight in kilograms by height in meters squared. Blood pressure was measured three times in supine position and the mean was calculated for this analysis. AF type was categorized as paroxysmal, persistent, or permanent according to the guidelines of the European Society of Cardiology (11).

### Brain Magnetic Resonance Imaging and Ischemic Brain Lesions

Brain magnetic resonance imaging (MRI) was performed using a 1.5 or 3 Tesla whole body scanner. The standardized acquisition protocol included a 3D T1 weighted (T1w) Magnetization Prepared Rapid Gradient Echo (MPRAGE) and a 2D axial fluid attenuated inversion recovery (FLAIR). Acquired MRI scans were transferred to an imaging clinical research organization specialized in international phase III pharmacological treatment trials (Medical Image Analysis Centre, Basel, Switzerland).

**Figure 1:**
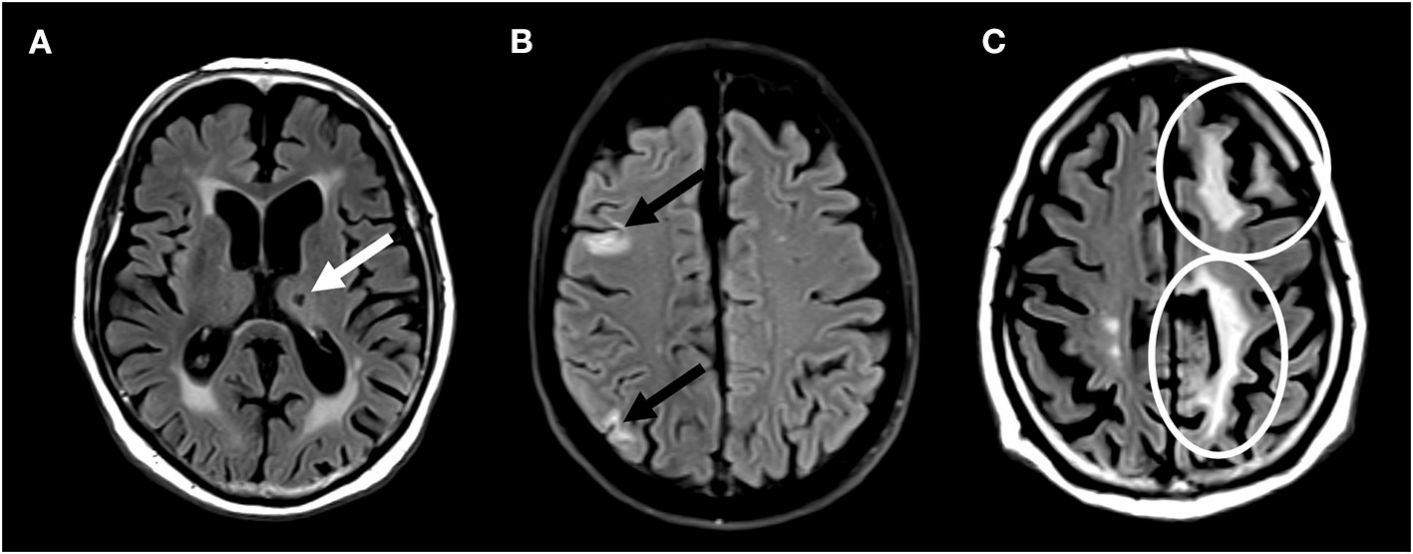
Brain magnetic resonance imaging showing **(A)** small non-cortical infarct **(B)** small cortical infarcts, and **(C)** large non-cortical and cortical infarcts.

Brain lesions were marked and segmented in a standardized fashion using Amira (Mercury Computer Systems Inc., Chelmsford, USA) by trained and validated raters and subsequently confirmed by expert readers. All investigators were blinded of the clinical evolution of the patients. Ischemic brain lesions were identified by their characteristic lesion morphology on both FLAIR and T1w images. Next, ischemic brain lesions were separated into small non-cortical infarcts or large non-cortical and cortical infarcts. Hereby, FLAIR-hyperintense lesions with low signal on T1w MPRAGE and a diameter of ≤ 20 mm, not involving the cortex, were defined as small non-cortical infarcts (SNCI). Possible localizations of SNCI were white matter, internal or external capsule, deep brain nuclei, thalamus and brainstem. Perivascular spaces were excluded by their tubular spatial appearance. Non-cortical infarcts with a diameter of >20 mm were defined as large non-cortical infarcts, whereas cortical infarcts were FLAIR-hyperintense lesions of any size, involving the cortex. Both ischemic lesion types are classified as large non-cortical and cortical infarcts (LNCCI) (4). Based on the MRI, the etiology of the ischemic lesion cannot be proven. However, it can be assumed, that LNCCI more likely occur due to embolic events, whereas SNCI are corresponding to microvascular brain damage. Any ischemic brain lesions were defined as either LNCCI or SNCI. Examples of SNCI, small cortical infarcts and LNCCI are presented in Figure 1.

### Outcome Events

The main outcome was the prevalence of ischemic brain lesions on MRI. “Clinically overt ischemic brain lesions” were defined as MRI-detected ischemic brain lesions in patients with a history of stroke. “Silent ischemic brain lesions” were defined as MRI-detected ischemic brain lesions in patients without a history of stroke or transient ischemic attack (TIA). “Any ischemic brain lesion” was defined as a composite of clinically overt and silent ischemic brain lesions. Since it was assumed that TIA does not cause MRI-detectable brain infarction, detected brain lesions in patients with a history of TIA were not classified as clinically overt or silent, but were considered in the composite outcome of any ischemic brain lesion (12).

### Adaption of the CHA_2_DS_2_-VASc Score

The main objective of this study was to investigate the association of the CHA_2_DS_2_-VASc score with clinically overt and silent ischemic brain lesions. For the purpose of this analysis, we therefore excluded the “S_2_” (history of stroke or TIA) component from the CHA_2_DS_2_-VASc score, and used an adapted CHA_2_D-VASc score comprising the following variables: history of heart failure, history of hypertension, age of 65–74 years and age ≥75 years, history of diabetes, vascular disease [including history of myocardial infarction, history of coronary artery disease, history of coronary artery bypass graft (CABG), history of percutaneous coronary intervention (PCI)/stenting, history of peripheral vascular disease] and female sex. Each variable corresponded to 1 point each, except age ≥75 years, which counted as 2 points. The total score ranged from 0 (low risk) to 7 (high risk). We used the CHA_2_D-VASc score for analyses with clinically overt, silent and any ischemic brain lesion. For analyses with the outcome of any ischemic brain lesion, we additionally used the conventional CHA_2_DS_2_-VASc score. In both scores we assigned 0 points to female patients who had no further risk factors (9). We grouped together patients with a CHA_2_D-VASc score ≥5, due to their small number in our cohort.

### Statistical Analysis

Baseline characteristics were stratified by the presence or absence of a history of stroke. Numbers are presented as counts (percentages) for categorical variables or means (± standard deviation) for continuous variables. Groups were compared using Chi-square tests for categorical variables, and Student's *t*-tests or Mann-Whitney-U-test for continuous variables, as appropriate. Prevalence of clinically overt and silent ischemic brain lesions was stratified according to the CHA_2_D-VASc score and presented as numbers (percentage).

To investigate the associations of the CHA_2_D-VASc score with ischemic brain lesions, we performed multivariable logistic regression analyses to calculate the odds ratio (OR) and corresponding 95% confidence interval (CI). Presence of clinically overt, silent and any ischemic brain lesions were used as binary outcome variables. For any ischemic brain lesion, additional analyses were performed using the CHA_2_DS_2_-VASc score. In addition, the association of the CHA_2_D-VASc score and the CHA_2_DS_2_-VASc score with LNCCI and SNCI was investigated using logistic regression models. All multivariable models were adjusted for BMI, smoking status, renal function, history of venous thromboembolism, history of systemic arterial embolism, AF type and intake of oral anticoagulation.

The goodness-of fit of the CHA_2_D-VASc score to predict clinically overt, silent or any ischemic brain lesions was compared based on the Akaike Information Criteria (AIC) and the Brier score. A similar approach was used for the CHA_2_DS_2_-VASc score to predict any ischemic brain lesion. The discriminative ability of both scores was assessed by calculating the c-statistic. We compared the predictive performance of different models, excluding each component of the CHA_2_D-VASc score in turn.

Furthermore, logistic regression analysis was performed to determine the association between each component of the CHA_2_D-VASc score and ischemic brain lesions. A combined model (model 1) was used, including all individual components of the CHA_2_D-VASc score. For the outcome of any ischemic brain lesion, we performed an additional analysis including all components of the CHA_2_DS_2_-VASc score in the combined model (model 2). Co-linearity in all combined models was evaluated using the variation inflation factor (VIF). Statistical analyses were performed using R Studio (Version 3.6.1).

## Results

Baseline characteristics are presented in Table 1. Mean age of this AF population was 73 ± 8 years, and 477 (27.4%) were female. The mean CHA_2_DS_2_-VASc score was 3.3 ± 1.7. Overall, 230 (13.2%) patients had a history of stroke. Patients with a history of stroke were older (75 ± 7 vs. 72 ± 9 years; *p* < 0.001), more frequently had hypertension (77.4 vs. 67.6%; *p* = 0.004), diabetes (21.3 vs. 14.7%; *p* = 0.01) and a history of bleeding (19.1 vs. 13.4%; *p* = 0.03) compared to patients without a history of stroke. The great majority of patients were anticoagulated, but patients with a history of stroke were more often on oral anticoagulation compared to patients without a history of stroke (94.3 vs. 89.5%; *p* = 0.03).

**Table 1:** Baseline characteristics stratified by presence or absence of history of stroke.

**Characteristic**	**All patients (*n* = 1,741)**	**Patients with history of stroke (*n* = 230)**	**Patients without history of stroke (*n* = 1,511)**	*P*-value^*^
Age, yrs	72.5 ± 8.4	74.9 ± 7.0	72.0 ± 8.6	<0.001
Female sex	477 (27.4%)	70 (30.4%)	407 (26.9%)	0.30
BMI, kg/m^2^	27.7 ± 4.8	27.7 ± 4.9	27.7 ± 4.7	0.95
Smoking status^†^				0.99
Current	132 (7.6%)	18 (7.8%)	114 (7.6%)	
Past	840 (48.3%)	111 (48.3%)	729 (48.3%)	
Never	767 (44.1%)	101 (43.9%)	666(44.1%)	
Systolic blood pressure^‡^, mm Hg	135 ± 19	135 ±19	135 ± 18	0.70
Diastolic blood pressure^‡^, mm Hg	78 ± 12	77 ± 12	79 ± 12	0.03
AF type				0.008
Paroxysmal	799 (45.9%)	175 (50.3%)	624 (44.8%)	
Persistent	524 (30.1%)	81 (23.3%)	443 (31.8%)	
Permanent	418 (24.0%)	92 (26.4%)	326 (23.4%)	
History of heart failure	378 (21.7%)	56 (24.3%)	322 (21.3%)	0.34
History of hypertension	1199 (68.9%)	178 (77.4%)	1021 (67.6%)	0.004
History of diabetes mellitus	271 (15.6%)	49 (21.3%)	222 (14.7%)	0.01
History of TIA	159 (9.1%)	41 (17.8%)	118 (7.8%)	<0.001
History of coronary heart disease	462 (26.5%)	67 (29.1%)	395 (26.1%)	0.38
History of peripheral vascular disease	122 (7.0%)	18 (7.8%)	104 (6.9%)	0.70
History of deep venous thrombosis/ pulmonary embolism	151 (8.7%)	22 (9.6%)	129 (8.5%)	0.97
History of bleeding^†^	246 (14.1%)	44 (19.1%)	202 (13.4%)	0.03
History of renal failure^§^	313 (18.0%)	48 (20.9%)	265 (17.5%)	0.26
CHA_2_DS_2_-VASc score	3.3 ± 1.7	5.3 ± 1.3	3.0 ± 1.6	<0.001
Oral anticoagulation	1,569 (90.1%)	217 (94.3%)	1,352 (89.5%)	0.03
Vitamin K antagonists (VKA)	634 (36.4%)	87 (37.8%)	547 (36.2%)	0.69
Direct oral anticoagulants (DOAC)	934 (53.6%)	130 (56.5%)	804 (53.2%)	0.38
Antiplatelet therapy^||^	306 (17.6%)	50 (21.9%)	256 (17.0%)	0.08
Previous pulmonary vein isolation	394 (22.6%)	24(10.4%)	370 (24.5%)	<0.001

*AF, atrial fibrillation; BMI, body-mass index; TIA, transient ischemic attack*.

*Data are presented as means (± standard deviation) or numbers (percentage), as appropriate*.

*CHA_2_DS_2_-VASc = congestive heart failure, hypertension, age ≥75 yrs (2 points), diabetes, prior stroke or TIA or thromboembolism (2 points), vascular disease, age 65–74 yrs, female sex*.

* *P-value compares the values between patients with or without history of stroke. Groups were compared using a Chi-squared test for categorical variables and Student's t-test or Mann-Whitney-U-test for continuous variables*.

† *n = 2 missings*,

‡ *n = 10 missings*,

§ *n = 1 missing*,

|| *n = 3 missings*.

**Figure 2:**
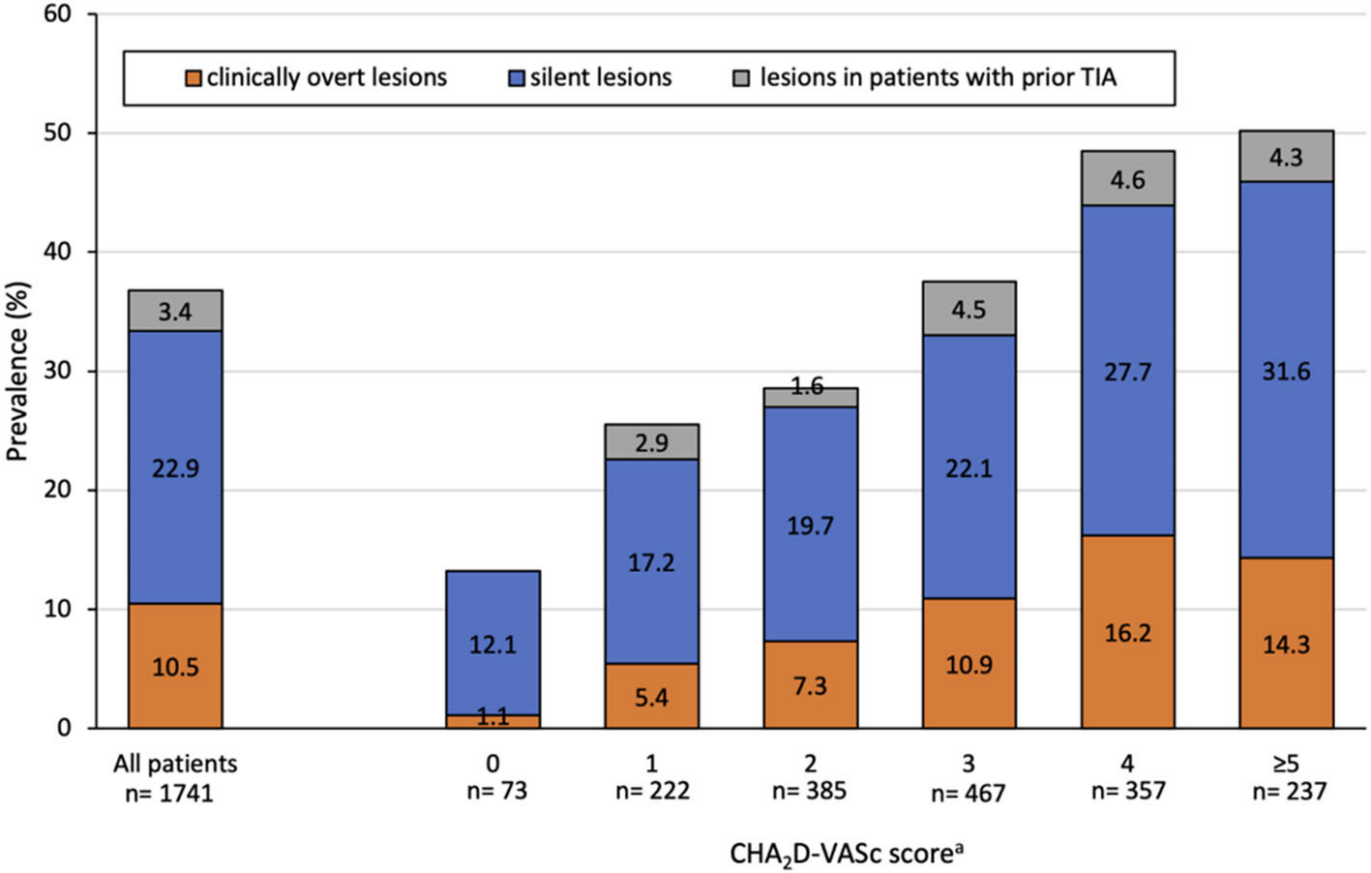
Prevalence of ischemic brain lesions stratified by the adapted CHA_2_D-VASc score. Abbreviations: TIA, transient ischemic attack. aExcluding history of stroke/TIA (S_2_). Female patients with a CHA_2_D-VASc score of 1 were assigned to the category of CHA_2_D-VASc score 0. Clinically overt ischemic brain lesions were defined as ischemic brain lesions in patients with a history of stroke. Silent ischemic brain lesions were defined as ischemic brain lesions in patients without a history of stroke/TIA.

The prevalence of ischemic brain lesions stratified by the CHA_2_D-VASc score is shown in Figure 2. Overall, 641 (36.8%) patients had at least one ischemic brain lesion. Of those, 399 (22.9%) were silent and 183 (10.5%) were clinically overt, whereby 59 (3.4%) patients with an ischemic brain lesion had a history of TIA. When distinguishing between the type of ischemic brain lesions, 390 (22.4%) had LNCCI and 373 (21.4%) had SNCI (Supplementary Table 1). The prevalence of ischemic brain lesions was increasing with a higher CHA_2_D-VASc score with the highest prevalence in patients with a risk score of ≥5.

Using the CHA_2_D-VASc score as an ordinal variable, we found linear relationships with clinically overt, silent and any ischemic lesions in a multivariable model (Table 2). Per 1 point increase in the adapted score, the multivariable adjusted OR (95% CI) for clinically overt ischemic brain lesions, silent ischemic brain lesions or any ischemic brain lesion were 1.32 (1.17–1.49; *p* < 0.001), 1.20 (1.10–1.30; *p* < 0.001) and 1.33 (1.23–1.44; *p* < 0.001), respectively. In each of the individual score categories of the CHA_2_D-VASc score, ORs were higher for clinically overt ischemic brain lesions compared to silent ischemic brain lesions. The results of the association of the CHA_2_D-VASc score with LNCCI and SNCI are presented in Supplementary Table 1. Per 1-point increase in CHA_2_D-VASc score, the OR (95% CI) for the presence of LNCCI or SNCI was 1.22 (1.12; 1.33), *p* < 0.001 and 1.37 (1.25; 1.50), *p* < 0.001.

**Table 2:** Relationship of the risk score with ischemic brain lesions.

	Adjusted Odds ratio (95% CI)^*^, ***p*****-value**		
	**Clinically overt ischemic brain lesions (*n* = 183)**	**Silent ischemic brain lesions (*n* = 399)**	**Any ischemic brain lesion (*n* = 641)**
CHA_2_D-VASc score^†^	1.32 (1.17, 1.49), *p* < 0.001	1.20 (1.10, 1.30), *p* < 0.001	1.33 (1.23, 1.44), *p* < 0.001
CHA_2_DS_2_-VASc score	NA	NA	1.52 (1.42, 1.62), *p* < 0.001
**CHA**_**2**_**D-VASc score^†^**			
≤ 1	1 (Reference)	1 (Reference)	1 (Reference)
2	1.76 (0.90, 3.67), *p* = 0.11	1.30 (0.87, 1.96), *p* = 0.21	1.40 (0.98, 2.01), *p* = 0.07
3	2.71 (1.46, 5.44), *p* = 0.003	1.49 (1.01, 2.21), *p* = 0.05	2.06 (1.47, 2.91), *p* < 0.001
4	4.15 (2.23, 8.35), *p* < 0.001	1.94 (1.30, 2.92), *p* = 0.001	3.07 (2.16, 4.41), *p* < 0.001
≥5	3.67 (1.85, 7.73), *p* < 0.001	2.22 (1.43, 3.46), *p* < 0.001	3.20 (2.16, 4.79), *p* < 0.001

*NA, not applicable*.

* *Multivariable models were adjusted for body mass index, smoking status, renal function, and history of deep venous thrombosis/pulmonary embolism, history of systemic embolism, atrial fibrillation type and oral anticoagulation, n = 3 missings*.

† *CHA_2_D-VASc score was adapted by excluding history of stroke/transient ischemic attack (S_2_)*.

*Female patients with a CHA_2_D-VASc score/CHA_2_DS_2_-VASc score of 1 were assigned to the category of 0 points*.

*Clinically overt ischemic brain lesions were defined as ischemic brain lesions in patients with a history of stroke*.

*Silent ischemic brain lesions were defined as ischemic brain lesions in patients without a history of stroke/TIA*.

*Any ischemic brain lesion was defined as a composite of clinically overt and silent ischemic brain lesions including ischemic lesions in patients with history of TIA*.

The predictive performance of the risk score for ischemic brain lesions is shown in Table 3. The C-statistic of the CHA_2_D-VASc score was 0.62 for clinically overt ischemic brain lesions and 0.58 for silent ischemic brain lesions. Compared to the CHA_2_DS_2_-VASc score, the CHA_2_D-VASc score had a lower C-statistic (0.63 vs. 0.69) and a higher AIC (2,217 vs. 2,099) and a higher Brier score (0.223 vs. 0.207) for predicting any ischemic brain lesion.

**Table 3:** Predictive performance of the risk score for ischemic brain lesions.

	CHA_**2**_D-VASc score^*^	**CHA_**2**_DS_**2**_-VASc score**
**Clinically overt ischemic brain lesions**		
C-statistic	0.622	NA
Brier Score	0.093	NA
AIC	1,147	NA
**Silent ischemic brain lesions**		
C-statistic	0.582	NA
Brier Score	0.174	NA
AIC	1,852	NA
**Any ischemic brain lesion**		
C-statistic	0.626	0.693
Brier Score	0.223	0.207
AIC	2,217	2,099

*AIC, Akaike's Information Criteria; NA, not applicable*.

* *CHA_2_D-VASc score was adapted by excluding history of stroke/transient ischemic attack (S_2_)*.

*Female patients with a CHA_2_D-VASc score/CHA_2_DS_2_-VASc score of 1 were assigned to the category of 0 points*.

*Clinically overt ischemic brain lesions were defined as ischemic brain lesions in patients with a history of stroke*.

*Silent ischemic brain lesions were defined as ischemic brain lesions in patients without a history of stroke/TIA*.

*Any ischemic brain lesion was defined as a composite of clinically overt and silent ischemic brain lesions including ischemic lesions in patients with history of TIA*.

Associations of the individual components of the CHA_2_D-VASc score and clinically overt, silent and any ischemic brain lesions are presented in Table 4. History of hypertension (OR 1.90; 95% CI 1.28–2.88; *p* = 0.002), age between 65 and 74 years (OR 2.58; 95% CI 1.29–5.90; *p* = 0.013), age ≥75 years (OR 4.13; 95% CI 2.07–9.43; *p* < 0.001) and history of diabetes (OR 1.48; 95% CI 1.00–2.18; *p* = 0.047) were associated with clinically overt ischemic brain lesions, but vascular disease, heart failure and female sex were not.

**Table 4:** Relationship of the individual components and ischemic brain lesions.

	**Clinically overt ischemic brain lesions (*****n*** **=** **183)**		**Silent ischemic brain lesions (*****n*** **=** **399)**		**Any ischemic brain lesion (*****n*** **=** **641)**		
	**No. of Cases**	**Odds ratio (95% CI)**	**No. of Cases**	**Odds ratio (95% CI)**	**No. of Cases**	**Odds ratio (95% CI)**	
**Components**		Model 1^*^		Model 1^*^		Model 1^*^	**Model 2^**†**^**
History of heart failure	46	0.96 (0.66, 1.39)	109	1.31 (0.99, 1.71)	168	1.19 (0.93, 1.53)	1.24 (0.96, 1.61)
History of hypertension	150	1.90 (1.28, 2.88)	279	0.87 (0.67, 1.12)	473	1.15 (0.92, 1.45)	1.11 (0.87, 1.42)
Age							
65–74 years^‡^	72	2.58 (1.29, 5.90)	161	1.95 (1.26, 3.10)	256	2.51 (1.72, 3.77)	2.16 (1.46, 3.29)
≥75 years^§^	103	4.13 (2.07, 9.43)	212	3.06 (1.98, 4.89)	350	4.71 (3.20, 7.10)	3.99 (2.67, 6.09)
History of diabetes	42	1.48 (1.00, 2.18)	69	0.98 (0.71, 1.34)	120	1.16 (0.87, 1.53)	1.09 (0.81, 1.46)
History of vascular disease	61	0.90 (0.63, 1.28)	153	1.39 (1.07, 1.79)	237	1.31 (1.04, 1.64)	1.30 (1.02, 1.65)
Female sex	52	0.99 (0.69, 1.41)	96	0.79 (0.60, 1.04)	165	0.84 (0.66, 1.06)	0.77 (0.60, 0.99)
History of stroke/TIA^||^					242		5.30 (4.08, 6.93)

*CI, confidence interval; TIA, transient ischemic attack*.

* *Model 1: The model was combined including all components of the CHA_2_D-VASc score (history of heart failure, history of hypertension, age 65–74 years and ≥75 years, history of diabetes, vascular disease and female sex)*.

† *Model 2: The model was combined including all components of the CHA_2_DS_2_-VASc score (history of heart failure, history of hypertension, age 65–74 years and ≥75 years, history of diabetes, vascular disease and female sex and history of stroke/TIA)*.

*Clinically overt ischemic brain lesions were defined as ischemic brain lesions in patients with a history of stroke*.

*Silent ischemic brain lesions were defined as ischemic brain lesions in patients without a history of stroke/TIA*.

*Any ischemic brain lesion was defined as a composite of clinically overt and silent ischemic brain lesions including ischemic lesions in patients with history of TIA*.

‡ *n = 789*,

§ *n = 722*,

|| *n = 348*.

Risk factors associated with silent ischemic brain lesions were age between 65 and 74 years (OR 1.95; 95% CI 1.26–3.10; *p* = 0.004), age ≥75 years (OR 3.06; 95% CI 1.98–4.89; *p* < 0.001) and history of vascular disease (OR 1.39; 95% CI 1.07–1.79; *p* = 0.012), but history of heart failure, hypertension, diabetes and female sex were not significantly associated with silent ischemic brain lesions. Risk factors associated with any ischemic brain lesion included age between 65 and 74 years (OR 2.51; 95% CI 1.72–3.77; *p* < 0.001), age ≥75 years (OR 4.71; 95% CI 3.20–7.10; *p* < 0.001) and history of vascular disease (OR 1.31; 95% CI 1.04–1.64; *p* = 0.026). However, history of heart failure, hypertension, diabetes and female sex were not significantly associated with any ischemic brain lesion. When adding history of stroke/TIA to the combined model (Model 2) for the outcome of any ischemic brain lesion, a strong association between history of stroke/TIA and any ischemic brain lesion was observed (OR 5.30; 95% CI 4.08–6.93; *p* < 0.001).

The impact of individual CHA_2_D-VASc components to the full CHA_2_D-VASc model is presented in Supplementary Table 2.

## Discussion

In this study investigating the associations between the CHA_2_D-VASc score and ischemic brain lesions in a cohort of well-characterized AF patients, the following main findings emerged: First, in this population of AF patients, the prevalence of ischemic brain lesions was high, and two thirds of all brain lesions were observed in patients without a history of stroke or TIA. Second, a higher CHA_2_D-VASc score was associated with a higher prevalence of ischemic brain lesions detected by MRI compared to lower scores. Third, the CHA_2_D-VASc score was associated not only with clinically overt but also with silent ischemic brain lesions. Fourth, the individual components of the CHA_2_D-VASc score showed slightly differential associations with clinically overt compared to silent ischemic brain lesions. Finally, both female sex and heart failure were not associated with clinically overt, silent or any ischemic brain lesions in any of the models.

In our study of mainly anticoagulated patients, the CHA_2_D-VASc score was linearly associated with the presence of silent and overt ischemic brain lesions. This is in line with the results of smaller studies in patients with aortic stenosis and low risk AF patients (13, 14). In addition, we found a strong association of the CHA_2_D-VASc score with the two different brain lesion categories of LNCCI and SNCI.

Increasing age, hypertension and diabetes mellitus are conventional risk factors for ischemic stroke (15). In our cohort, we found similar associations. Age of ≥ 65 years was an important risk factor not only for clinically overt, but for silent ischemic brain lesions as well, which is in line with findings from other epidemiologic studies (8, 14, 16).

We have found that the presence of vascular disease was associated with silent ischemic brain lesions. Prior studies have suggested that cerebral small vessel disease plays an important role in the pathogenesis of silent brain infarcts (17, 18). Cerebral small vessel disease is defined as segmental atherosclerotic disorganization of the small perforating arteries, which leads to a process of ischemia and infarction, resulting in tissue damage and lacunar scar formation (18). The vascular disease burden in our cohort may suggest coexisting small vessel disease, which in turn is associated with silent ischemic brain lesions. In addition, hypertension, considered one of the most important causes of cerebral small vessel disease (18), was a strong predictor for silent ischemic brain lesions in several studies (16, 19). Interestingly, in our cohort, although associated with clinically overt ischemic brain lesions, hypertension was not associated with silent brain lesions. This finding may be explained by the fact that measured systolic blood pressure in our cohort was nearly 20 mmHg lower compared to other study populations (20, 21)

Female sex was neither associated with clinically overt nor with silent ischemic brain lesions in our cohort, which is in contrast to other reports suggesting that women have a higher risk of (overt) stroke than men (15, 22). Regarding silent ischemic brain lesions, analyses from the Atherosclerosis Risk in Communities Study showed that female sex was not related to an increased risk of silent brain infarcts (23). Similarly, in the Rotterdam Scan Study, female sex was associated with silent ischemic brain lesions in age and sex adjusted models, but not after adjustments for additional covariates (20). Our results suggest that risk stratification for stroke using the CHA_2_DS_2_-VASc scheme without female sex as a risk factor is debatable, since female sex may be considered as a risk modifier rather than a risk factor in stroke risk assessment (24). Given the heterogeneity of study results on sex as a potential independent risk factor, further research is needed to identify potential sex-specific differences in the development of silent brain infarcts (20, 23).

Neither clinically overt nor silent ischemic brain lesions were associated with a history of heart failure in our population. This interrelationship has been investigated in recent studies, suggesting that heart failure *per se* may not be a strong predictor for clinically overt ischemic brain lesions (8, 15). These findings are also supported by the results of a recent trial showing that patients with diagnosed heart failure, albeit without known AF, do not benefit from oral anticoagulation for stroke prevention (25).

In clinical practice, the CHA_2_DS_2_-VASc score is used for risk assessment with the purpose of the prevention of stroke, whereas a history of stroke or TIA in AF patients corresponds to 2 points according to the CHA_2_DS_2_-VASc scheme, these patients qualify for secondary prevention using oral anticoagulation independent of their stroke risk based on the CHA_2_DS_2_-VASc score. Therefore, regarding primary prevention, excluding the “history of stroke” component from the score could be considered, as it artificially increases the apparent discriminative ability to predict ischemic brain lesions. Since MRI was performed in this study, providing information on clinically overt and silent brain infarcts, we performed risk stratification using a CHA_2_D-VASc score and analyzed its predictive value for clinically overt, silent and any ischemic brain lesion. The modest reported C-statistics show that additional tools for better risk prediction would be desirable. Stroke prediction can be improved slightly by additional use of biomarkers, but whether a biomarker score guided approach improves outcomes is unknown but currently tested in a randomized trial (26).

Our findings also underscore the importance of gaining knowledge in specific causes for silent ischemic brain lesions in patients with AF. Currently, we might underestimate the risk of silent ischemic strokes in AF patients. As the prevalence of silent ischemic brain lesions is shown to be associated with a higher risk for future symptomatic stroke, a screening tool for the detection of silent ischemic brain lesions might be justified to evaluate stroke risk even in lower risk AF patients (5, 6). However, the clinical value of such an approach would need to be tested in a prospective study. Additionally, given that silent ischemic brain lesions are independently associated with cognitive dysfunction, identifying additional factors beyond the CHA_2_DS_2_-VASc score that may contribute to the development of these lesions is imperative (4).

## Strengths and Limitations

The major strengths of our study are the large and well-characterized community-based cohort of AF patients and the very low rate of missing values. Additionally, brain MRIs were acquired and validated centrally using standard operating procedures. However, some potential limitations need to be considered. First, given the observational design of the study, causality of the associations cannot be inferred. Second, clinical presentation of previous stroke was not systematically evaluated. Therefore, we could not distinguish which lesions were actually associated with the overt stroke and which were silent in patients with multiple ischemic brain lesions and a positive stroke history. Third, our cohort does not represent a treatment-naïve patient group because the vast majority of patients were on oral anticoagulation. However, it also reflects the level of care in a country with a high standard in healthcare and therefore most likely daily practice in developed countries. Finally, due to the study design, it could not be determined whether the ischemic brain lesion occurred before or after anticoagulation was started.

## Conclusion

In conclusion, we found that an increasing CHA_2_D-VASc score was associated with a higher prevalence of ischemic brain lesions in AF patients. The predictive value of the CHA_2_D-VASc score was modest both for clinically overt and silent ischemic brain lesions. Differential associations of the individual components of the score were observed for clinically overt compared to silent ischemic brain lesions.

## Data Availability Statement

Due to restrictions by the ethical committee, data is not publicly available. Requests to access the datasets should be directed to michael.kuehne@usb.ch.

## Ethics Statement

The studies involving human participants were reviewed and approved by Ethikkomission Nordwest- und Zentralschweiz. The patients/participants provided their written informed consent to participate in this study.

## Author Contributions

All authors listed have made a substantial, direct and intellectual contribution to the work, and approved it for publication.

## Conflict of Interest

MK has received grants from the Swiss National Science Foundation, the Swiss Heart Foundation, Bayer and Pfizer-BMS, he has received lecture/consulting fees from Daiichi-Sankyo, Boehringer Ingelheim, Bayer, Pfizer-BMS, AstraZeneca, Sanofi-Aventis, Novartis, MSD, Medtronic, Boston Scientific, St. Jude Medical, Biotronik, Sorin, Zoll, and Biosense Webster. DC received consulting fees from Servier, Canada, outside of the presented work. NR has received a grant from the Swiss Heart Foundation. LB has received grants from the Swiss National Science Foundation, the University of Basel, the Swiss Heart Foundation, The Stroke Association, and AstraZeneca; and has received consulting and advisory board fees from Amgen, Bayer, Bristol-Myers Squibb, and Claret Medical. CC has received grants from the Swiss Heart Foundation and advisory board fees from Bayer, Medtronic, Boehringer Ingelheim and Pfizer. TR has received research grants from the Goldschmidt-Jacobson Foundation, the Swiss National Science Foundation, the Swiss Heart Foundation, the European Union, the Professor Max Cloëtta Foundation, the Cardiovascular Research Foundation Basel, the University of Basel and the University Hospital Basel, all outside of the presented work. He has received speaker/consulting honoraria or travel support from Abbott/SJM, Astra Zeneca, Brahms, Bayer, Biosense-Webster, Medtronic, Pfizer-BMS and Roche, all outside of the presented work. He has received support for his institution's fellowship program (Inselspital Bern) from Biosense-Webster, Biotronik, Medtronic, Abbott/SJM and Boston Scientific, all outside of the presented work. MB has received a grant from the Swiss National Science Foundation. The remaining authors declare that the research was conducted in the absence of any commercial or financial relationships that could be construed as a potential conflict of interest.
