# Supplementary material for: Association of the CHA2D(S2)-VASc Score and Its Components With Overt and Silent Ischemic Brain Lesions in Patients With Atrial Fibrillation
Source: Front Neurol. 2021 Jan 12;11:609234. doi: 10.3389/fneur.2020.609234 (PMC7835704; doi:10.3389/fneur.2020.609234)
Supplement: Supplementary file 1 [file Data_Sheet_1.docx]

**Supplement**

**Association of the CHA_2_D(S_2_)-VASc Score and its Components with Overt and Silent Ischemic Brain Lesions in Patients with Atrial Fibrillation**

Fabienne Steiner, Pascal Meyre, Stefanie Aeschbacher, Michael Coslovsky, Tim Sinnecker, Manuel R Blum, Nicolas Rodondi, Carlo W Cereda, Marcello di Valentino, Florence Wenger, Andrea Cussigh, Philipp Krisai, Laurent Roten, Tobias Reichlin, David Conen, Stefan Osswald, Leo H Bonati, Michael Kühne, on behalf of the Swiss-AF Investigators

**Figure S1** Flow diagram of patient inclusion

**Table S1** Relationship of the risk score with large non-cortical and cortical infarcts (LNCCI) and small non-cortical infarcts (SNCI)

**Table S2** Impact of individual risk factors to the full risk score

**Supplemental Figure S1.** Flow diagram of patient inclusion


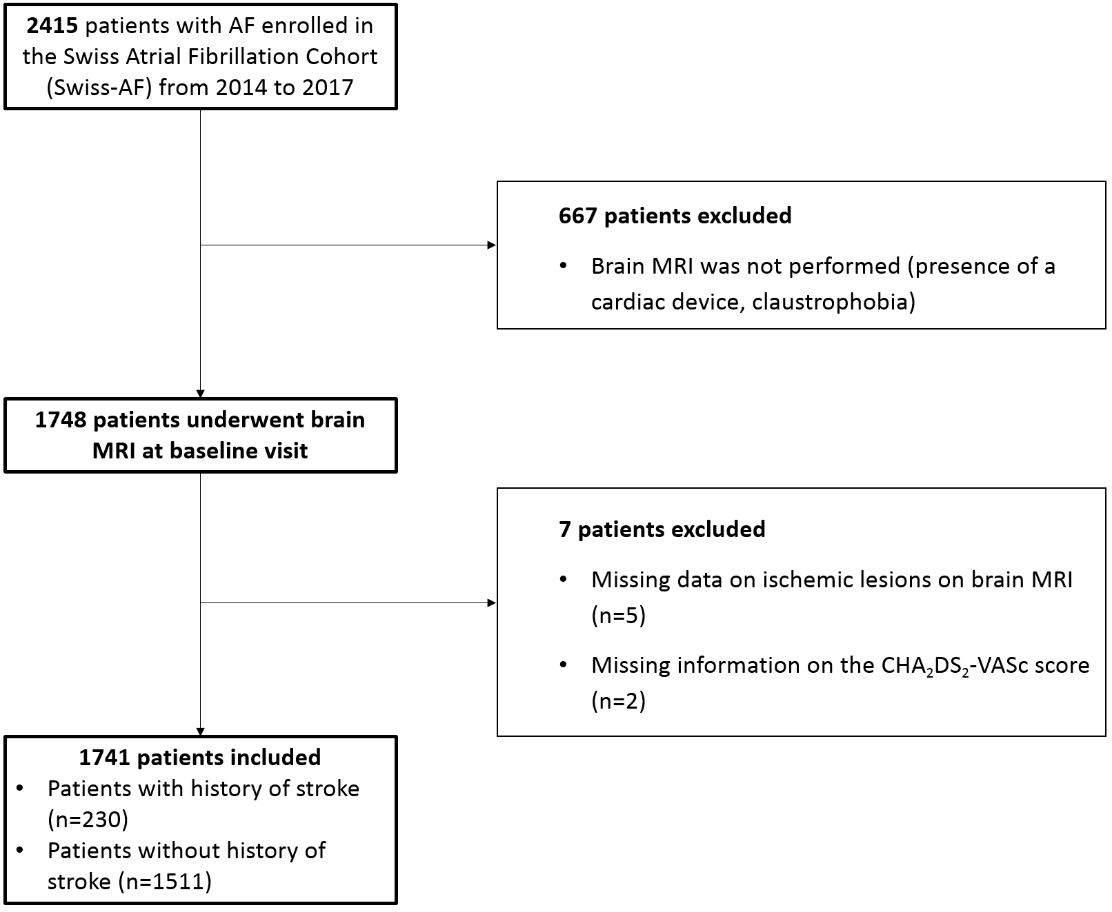


**Supplemental Table S1.** Relationship of the risk score with large non-cortical and cortical infarcts (LNCCI) and small non-cortical infarcts (SNCI)

|  | **Adjusted Odds ratio (95% CI) ^*^, p-value** | | | |  |
| --- | --- | --- | --- | --- | --- |
|  | **LNCCI**  **(n=390)** |  | **SNCI**  **(n=373)** |  | |
| **CHA_2_D-VASc score** ^†^ | 1.22 (1.12; 1.33), p<0.001 |  | 1.37 (1.25; 1.50), p<0.001 |  | |
| **CHA_2_DS_2_-VASc score** | 1.50 (1.40; 1.62), p<0.001 |  | 1.39 (1.29; 1.49), p<0.001 |  | |
| **CHA_2_D-VASc score** ^†^ |  |  |  |  | |
| **≤1** | 1 [Reference] |  | 1 [Reference] |  | |
| **2** | 1.09 (0.71; 1.69), p=0.69 |  | 2.02 (1.26; 3.31), p=0.004 |  | |
| **3** | 1.80 (1.22; 2.71), p=0.004 |  | 2.86 (1.84; 4.60), p<0.001 |  | |
| **4** | 2.37 (1.58; 3.60), p<0.001 |  | 3.85 (2.44; 6.25), p<0.001 |  | |
| **≥5** | 2.03 (1.28; 3.23), p=0.003 |  | 4.56 (2.78; 7.65), p<0.001 |  | |
| Abbreviations: LNCCI, large non-cortical and cortical infarcts; SNCI, small non-cortical infarcts.  ^*^ Multivariable models were adjusted for BMI, smoking status, renal function, and history of deep venous thrombosis/pulmonary embolism, history of systemic embolism, AF type and oral anticoagulation. n=3 missings.  ^†^ CHA_2_D-VASc score was adapted by excluding history of stroke/transient ischemic attack (S_2_).  Female patients with a CHA_2_D-VASc score/CHA_2_DS_2_-VASc score of 1 were assigned to the category of 0 points. | | | | | |

**Supplemental Table S2.** Impact of individual risk factors to the full risk score

|  | **CHA_2_D-VASc Models*** | | | | | | |
| --- | --- | --- | --- | --- | --- | --- | --- |
|  | Full model | Excluding history of heart failure | Excluding history of hypertension | Excluding age | Excluding history of diabetes | Excluding history of vascular disease | Excluding female sex |
| **Clinically overt ischemic brain lesions** | | | | | | | |
| Odds ratio (95%CI) | 1.34  (1.20, 1.49)  p< 0.001 | 1.42  (1.25, 1.61)  p< 0.001 | 1.32  (1.17, 1.50)  p< 0.001 | 1.30  (1.13, 1.5)  p< 0.001 | 1.35  (1.20, 1.53)  p< 0.001 | 1.41  (1.25,1.60)  p< 0.001 | 1.35  (1.21, 1.51)  p< 0.001 |
| C- statistic | 0.622 | 0.622 | 0.608 | 0.590 | 0.611 | 0.628 | 0.621 |
| Brier score | 0.093 | 0.093 | 0.093 | 0.093 | 0.093 | 0.093 | 0.093 |
| AIC | 1147 | 1144 | 1154 | 1161 | 1150 | 1144 | 1146 |
| **Silent ischemic brain lesions** | | | | | | | |
| Odds ratio  (95% CI) | 1.23  (1.14, 1.33)  p< 0.001 | 1.24  (1.14, 1.36)  p< 0.001 | 1.30  (1.19, 1.42)  p< 0.001 | 1.16  (1.05,1.29)  p< 0.001 | 1.27  (1.16, 1.38)  p< 0.001 | 1.21  (1.11,1.33)  p< 0.001 | 1.28  (1.18, 1.39)  p< 0.001 |
| C- statistic | 0.582 | 0.575 | 0.591 | 0.544 | 0.585 | 0.567 | 0.594 |
| Brier score | 0.174 | 0.174 | 0.173 | 0.176 | 0.174 | 0.175 | 0.173 |
| AIC | 1852 | 1856 | 1846 | 1870 | 1850 | 1860 | 1844 |
| **Any ischemic brain lesion** | | | | | | | |
| Odds ratio  (95% CI) | 1.37  (1.28, 1.47)  p< 0.001 | 1.42  (1.31, 1.55)  p< 0.001 | 1.43  (1.32, 1.56)  p< 0.001 | 1.28  (1.17,1.40)  p< 0.001 | 1.41  (1.31, 1.53)  p< 0.001 | 1.21  (1.11, 1.33)  p< 0.001 | 1.42  (1.32, 1.54)  p< 0.001 |
| C- statistic | 0.625 | 0.621 | 0.625 | 0.577 | 0.625 | 0.615 | 0.633 |
| Brier score | 0.223 | 0.223 | 0.223 | 0.228 | 0.222 | 0.224 | 0.221 |
| AIC | 2217 | 2218 | 2217 | 2266 | 2216 | 1860 | 2204 |
| Abbreviations: AIC, Akaike’s Information Criteria; CI, confidence interval.  *CHA_2_D-VASc score was adapted by excluding history of stroke/TIA (S_2_).  Clinically overt ischemic brain lesions were defined as ischemic brain lesions in patients with history of stroke.  Silent ischemic brain lesions were defined as ischemic brain lesions in patients without history of stroke or transient ischemic attack (TIA).  Any ischemic brain lesion was defined as a composite of clinically overt and silent ischemic brain lesions including ischemic lesions in patients with history of TIA. | | | | | | | |

**Swiss-AF investigators**

University Hospital Basel and Basel University: Stefanie Aeschbacher, Chloe Auberson, Steffen Blum, Leo Bonati, Selinda Ceylan, David Conen, Simone Doerpfeld, Ceylan Eken, Marc Girod, Peter Hämmerle, Philipp Krisai, Michael Kühne, Christine Meyer-Zürn, Pascal Meyre, Andreas U. Monsch, Christian Müller, Stefan Osswald, Philipp Reddiess, Anne Springer, Fabienne Steiner, Christian Sticherling, Thomas Szucs, Gian Voellmin, Leon Zwimpfer.

Principal Investigator: Stefan Osswald; Local Principal Investigator: Michael Kühne

University Hospital Bern: Faculty: Drahomir Aujesky, Urs Fischer, Juerg Fuhrer, Laurent Roten, Simon Jung, Heinrich Mattle; Research fellows: Luise Adam, Carole Elodie Aubert, Martin Feller, Claudio Schneider, Axel Loewe, Elisavet Moutzouri; Study nurses: Tanja Flückiger, Cindy Groen, Damiana Rakovic, Rylana Wenger, Lukas Ehrsam, Alexandra Nuoffer, Nathalie Schwab. Local Principal Investigator: Nicolas Rodondi

Stadtspital Triemli Zurich: Christopher Beynon, Roger Dillier, Michèle Deubelbeiss, Franz Eberli, Christine Franzini, Isabel Juchli, Claudia Liedtke, Jacqueline Nadler, Thayze Obst, Noreen Tynan, Xiaoye Schneider, Katrin Studerus, Dominik Weishaupt. Local Principal Investigator: Andreas Müller

Kantonspital Baden: Simone Fontana, Silke Kuest, Karin Scheuch, Denise Hischier, Nicole Bonetti, Alexandra Grau, Jonas Villinger, Eva Laube, Philipp Baumgartner, Mark Filipovic, Marcel Frick, Giulia Montrasio, Stefanie Leuenberger, Franziska Rutz. Local Principal Investigator: Jürg-Hans Beer

Cardiocentro Lugano: Angelo Auricchio, Adriana Anesini, Cristina Camporini, Giulio Conte, Maria Luce Caputo, Francois Regoli. Local Principal Investigator: Tiziano Moccetti

Kantonsspital St. Gallen: Roman Brenner, David Altmann, Michaela Gemperle. Local Principal Investigator: Peter Ammann

Hôpital Cantonal Fribourg: Mathieu Firmann, Sandrine Foucras. Local Principal Investigator: Daniel Hayoz

Luzerner Kantonsspital: Benjamin Berte, Virgina Justi, Frauke Kellner-Weldon, Brigitta Mehmann, , Myriam Roth, Andrea Ruckli-Kaeppeli, Ian Russi, Kai Schmidt, Mabelle Young, Melanie Zbinden. Local Principal Investigator: Richard Kobza

Ente Ospedaliero Cantonale Lugano: Jane Frangi-Kultalahti, Anica Pin, Luisa Vicari Local Principal Investigator: Giorgio Moschovitis

University Hospital Geneva: Georg Ehret, Hervé Gallet, Elise Guillermet, Francois Lazeyras, Karl-Olof Lovblad, Patrick Perret, Philippe Tavel, Cheryl Teres. Local Principal Investigator: Dipen Shah

University Hospital Lausanne: Nathalie Lauriers, Marie Méan, Sandrine Salzmann. Local Principal Investigator: Jürg Schläpfer

Bürgerspital Solothurn: Andrea Grêt, Jan Novak, Sandra Vitelli. Local Principal Investigator: Frank-Peter Stephan

Ente Ospedaliero Cantonale Bellinzona: Jane Frangi-Kultalahti, Augusto Gallino. Local Principal Investigator: Marcello Di Valentino

University of Zurich/University Hospital Zurich: Fabienne Witassek, Matthias Schwenkglenks.

Medical Image Analysis Center AG Basel: Jens Würfel (Head), Anna Altermatt, Michael Amann, Petra Huber, Esther Ruberte, Tim Sinnecker, Vanessa Zuber.

Clinical Trial Unit Basel: Michael Coslovsky (Head), Pascal Benkert, Gilles Dutilh, Milica Markovic, Patrick Simon

Schiller AG Baar: Ramun Schmid
